# Critical medical humanities: embracing entanglement, taking risks

**DOI:** 10.1136/medhum-2015-010692

**Published:** 2015-06

**Authors:** William Viney, Felicity Callard, Angela Woods

**Affiliations:** 1 Department of English Studies and Centre for Medical Humanities, Durham University, Durham, UK; 2 Department of Geography and Centre for Medical Humanities, Durham University, Durham, UK; 3 School of Medicine, Pharmacy and Health, and Centre for Medical Humanities, Durham University, Durham, UK

**Keywords:** Social science

## Abstract

What can the medical humanities achieve? This paper does not seek to define what is meant by the medical humanities, nor to adjudicate the exact disciplinary or interdisciplinary knowledges it should offer, but rather to consider what it might be capable of doing. Exploring the many valences of the word ‘critical’, we argue here for a critical medical humanities characterised by: (i) a widening of the sites and scales of ‘the medical’ beyond the primal scene of the clinical encounter; (ii) greater attention not simply to the context and experience of health and illness, but to their constitution at multiple levels; (iii) closer engagement with critical theory, queer and disability studies, activist politics and other allied fields; (iv) recognition that the arts, humanities and social sciences are best viewed not as in service or in opposition to the clinical and life sciences, but as productively entangled with a ‘biomedical culture’; and, following on from this, (v) robust commitment to new forms of interdisciplinary and cross-sector collaboration. We go on to introduce the five other articles published in this special issue of the journal, reflecting on the ways in which collaboration and critique are articulated in their analyses of immunology, critical neuroscience, toxicity, global clinical labour, and psychological coercion and workfare. As these articles demonstrate, embracing the complex role of critical collaborator—one based on notions of entanglement, rather than servility or antagonism—will, we suggest, develop the imaginative and creative heterodox qualities and practices which have long been recognised as core strengths of the medical humanities.

## What can a critical medical humanities achieve?

Clinicians, researchers, healthcare workers, artists and many others who routinely engage with or contribute to the medical humanities may despair that this is yet another attempt to define the medical humanities,^1–4^ whether it is taken to be a discipline or a field of enquiry, or as a set of interventions, shared values, or interdisciplinary collaborative relationships. This special issue encompasses the many valences of the word ‘critical’—urgent, sceptical, evaluative, and mobilising the long philosophical and political traditions of critique^5^—to discover not what is meant by the medical humanities, nor to adjudicate the exact disciplinary or interdisciplinary knowledges it should offer, but rather to explore what it is capable of doing. If diversity and plurality have, in the past, traditionally been strengths for the medical humanities in terms of encouraging creativity and epistemological innovation, then this collection of papers and responses is intended as an invitation to keep the field of medical humanities open to new voices, challenges, events, and disciplinary (and anti- or post-disciplinary) articulations of the realities of medicine and health; to be adventurous in its intellectual pursuits, practical activities, and articulation with the domain of the political.

This does not mean abandoning what has given the medical humanities its successes, not least its resistance to positivist biomedical ‘reductionism’,^1^ its sensitivity to narrative-based interventions and their limitations,^6–11^ its designation of the patient–clinician relation as a renewed focus of attention,^12^ its interest in concepts of disease and practices of diagnosis,^13–15^ the dynamic role of the arts in health,^16^ and the therapeutic importance of comparative histories.^17^ ^18^ But, we argue, it does involve actively reflecting upon and interrogating the normative and individualist restrictions that may accompany these strategic gains. We are thinking, for example, of the frequency with which some of those aforementioned areas of focus have been enabled by particular—humanist—models of the self, of the ill and suffering body, and of modes of intervention and care. They have also been (too) often characterised by a dogged focus on the limitations of biomedical knowledge, and on how the humanities might bring empathy to clinical practice if allowed adequate epistemological space. Contributions to this special issue disrupt those models and the implicit disciplinary responsibilities that underpin them: they emerge from fields and areas of concern (including Continental philosophy, science and technology studies, activist politics, queer theory and disability studies) that, while proximate to the usual terrain of the medical humanities, have not always been visible to them; they are eclectic in the sites in which and scales at which enquiries are focused; and they all confront the uneven global terrain of health and medical care and policy, industry and scientific research.^19^ In addition, this special issue positions the medical humanities as a powerful tool through which to address not only the meaning and historico-cultural *contexts* of health and illness, but their very production, concrescence and dispersal across the precarious, unequal and environmentally degraded societies in which we live.

In this introductory paper, we briefly open out some of the theoretical debates in which we wish to intervene: the practice of medical humanities scholarship, the position of the medical humanities in contemporary society, its relationship with predominant biomedical understandings of health and well-being, its assumed intimacy with certain disciplines over others, and the importance of collaborative models for creating new resources, methods and kinds of evidence. We call for more intensive engagement in the medical humanities with how health, illness and treatment are constituted in and through tangled webs of human and non-human biosocial organisms, political-economic formations, discourses and affects. Can the medical humanities intervene more explicitly in *ontological* questions—in particular, of aetiology, pathogenesis, intervention and cure—rather than, as has commonly been the case, leaving such questions largely to the domains of the life sciences and biomedicine? To think further about such questions demands not a manifesto but an attempt to amplify and magnify a set of existing imperatives,^20–22^ a clarion call for a richer, deeper, more intense debate about what medical humanities can achieve.

## ‘Critical’ what?

Twentieth and 21st century thought, especially when generated in European and US universities, has seen many turns to and with ‘the critical’ as a rallying point for a new bloc or grouping of politically committed researchers. Of these, the philosophical scepticism and political activism of the Frankfurt School, and its commitment to critical theory as a means to form an intellectual community and bring about social change, have been among the most influential. The work of Theodor Adorno, Max Horkheimer and Hannah Arendt, among others, built on a much longer European tradition of written *critique*, of sustained and methodical analysis of a given object or process. Equally important to the formation of a critical character within both the humanities and social sciences has been the genealogical work of Michel Foucault, whose research into expressions of authority has refused processes of humanistic naturalisation and its relationship to government, stressing the importance of critique as a means to resist ‘presumptuous reason and its specific effects of power’.^23^

Just as sociology, geography, law, public health and literary studies has each had a ‘critical turn’, marked by explicit attempts to reflect upon the underlying suppositions that ground the knowledge it produces, commonly attended by diverse expressions of political commitment, we recognise a need to reflect upon the given norms, procedures and values of our medical humanities research community. These include but are not exhausted by how ‘race’ and ethnicity, sexuality and gender, disability (and madness), technology and media, economics, and social and environmental inequalities are central to the production of medical knowledge and to the experience of health and illness. Our advocacy for a reflexively *critical* stance is not in the service of a particular political agenda or particular epistemological priorities, or, indeed, in favour of a precise programme of reform. Rather, we are more interested in illuminating diverse ways of doing medical humanities that are not only sensitive to imbalances of power, implicit and explicit, but include activist, sceptical, urgent and capacious modes of making and re-making medicine (and those domains closely allied to it)—and hence its ability to transform, for good and ill, the health and well-being of individuals and societies. Here, we are indebted to Judith Butler's characterisation of critique not simply as ‘the practice of destruction, of nay-saying, of nihilism, or of unbridled skepticism’; for if, as Butler argues, critique might facilitate ‘revolution at the level of procedure without which we cannot secure rights of dissent and processes of legitimation’,^24^ then we need to consider what and how both medicine and the medical humanities come to legitimate particular epistemological projects as central, and foreclose other potential projects from coming into view. It is our hope that this special issue will bring to light some of the restrictions that might mark the medical humanities at the level of procedure, and, simultaneously, function as critique by opening the possibility of revolution at that level.

Here, ‘critical medical humanities’ describes tendencies that are by no means new to or easily achieved by the medical humanities. The phrase recognises and gives a name to responsibilities that we wish to promote, but we in no way wish to become gatekeepers to the critical; indeed, it is precisely a proprietorial attitude that we hope a sharpened critical spirit can warn against. We believe that a critical medical humanities will flourish wherever the procedural norms and routines of the humanities, the social sciences, *and* the biological sciences are openly, evenly and creatively interrogated and reworked. This means admitting biases and challenging authorising discourses that have limited our understandings of what health and illness might be; it also, we suggest, implies resisting strategies of critique that rely simply on inter-discipline condemnation. In so doing, a framework in which the ‘perspectives’ of the humanities are pitted against those of the ‘sciences’ or ‘social sciences’ might give way to a much richer and more entangled investigation of the bio-psycho-social-physical events that underpin the life, and death, of any organism.

## Against role-play

We call, then, for an intensification of medical humanities’ critical engagement with the norms and routines that have, in the past, given it a ‘role’ within medicine and subsequently defined its arena of operation. We would like to dislodge the two common narratives of purpose that are attached to the medical humanities and challenge the units of identity and community that they tend to mobilise. First, there is a service or utilitarian model, which accommodates but does not actively seek to challenge pre-existing power structures and epistemological divisions of labour within biomedicine, and which claims to ‘improve the quality of the humane relationship among doctors, clinical professionals and patients’^25^ by becoming ‘a boon companion or supportive friend’^26^ to biomedical science. Humanising the objectivity of biomedicine, the medical humanities is felt to be ‘called upon to play a role in education and practice’.^27^ Positive, pliant and benevolent, two complementary cultures are understood to work collectively towards the smooth and optimal provision of medical or health programmes.

In contrast to this servile vision of the medical humanities, but no less dominated by the expectation that it should have a ‘role’ to play, others have defined its work according to its capacity to disrupt, broaden and embellish what are taken to be the overly reductive, materialist and scientistic definitions of human experience promoted by biomedicine.^28^ The medical humanities is presented as a counterbalance to the restrictive and restricted views of science. Without necessarily rejecting the body's materialities, it queries how, as Bethan Evans has written, ‘some bodies are seen as more equal than others’.^29^ This variety of medical humanities explores and expands views of human health, well-being and illness through subjective testimony, frequently re-evaluating whose knowledge counts within clinical encounters by collaborating with and including the analytic judgements of non-medical practitioners.^30^ ^31^ A different set of self-describing roles, with their own narrative trajectories, come to the fore—a medical humanities imagined to be antagonistic, noisy and opinionated; less a ‘supportive friend’ to biomedicine than a ‘disruptive teenager’.^32^ This follows what Bruno Latour sees as the traditional form of post-war critique, seeking to debunk and demystify, ‘to detect the real prejudices hidden behind the appearance of objective statements’.^33^ In a position of heroic rebellion, the medical humanities can be said to harness ‘the intellectual practice of the humanities with all of its democratising energies and dangerous possibilities, which enable and promote fearless questioning of representations, challenges to the abuses of authority and a steadfast refusal to accept as the limits of enquiry the boundaries that medicine sets between biology and culture’.^34^ This version of medical humanities is hostile, dogged, sceptical, and separable from the medical practices it seeks to target.

Within these established and predetermined critical pathways, which have sought to embolden a sense of tradition and responsibility within the medical humanities—either in service or in rejection of a ‘biomedical culture’—we believe a critical medical humanities should hesitate before assuming either a conciliatory or iconoclastic mandate as regards agents thought to be exterior to it. Assuming the practice of knowledge production and of medical care and healthcare to be static, the pitched battle overlooks how relationships between different practitioners can be conciliatory *and* antagonistic, and how there can indeed be, within one practitioner or set of practices, diverse enactments of the so-called ‘boundaries that medicine sets between biology and culture’.^30^ We are wary of the manner in which both trajectories establish a strong set of established allies and antagonists, and become absorbed in forms of disciplinary and/or expertise-based competition that gainsay the heterogeneous practices and epistemologies that undoubtedly characterise most fields.^35^ Many actors who populate the medical humanities are, we should recall, specialist multi-taskers: they collaborate across and between disciplines, inside and outside of clinical and para-clinical spaces, and frequently move from the position of patient to clinician to researcher to future patient. In such movements are born new practices and alliances that course across methodologies, epistemologies, kinds of experimental space and design. Rather than patrolling the ‘critical’ in the hope of creating a new orthodoxy, it is better, in our view, to celebrate and develop the imaginative and creative heterodox qualities and practices that have long been recognised as a core strength of the medical humanities.^36^

It is worth reminding ourselves of what Stephen Pattison wrote in this journal more than 10 years ago, where he argued that the medical humanities is threatened by ‘routinisation, exclusivism, narrowing, specialisation, professionalisation’ whenever as a field it ‘excludes varieties of disciplinary perspective and performance, has qualified exponents who are experts and who do not need to consult people in other disciplines, and becomes an autonomous discipline in its own right which licenses it [sic] own practitioners in some way’.^37^

The critical challenge facing the medical humanities comprises those kinds of creative and relational enclosure, even if that enclosure arises from a liberal critique of the powerful presented from a place of benevolent neutrality. Rather than embrace a territorialised conception of the medical humanities—one in which a vaguely defined community is said to occupy, defend and advance a ‘domain’ or ‘field’ in the face of some real or imagined combatant—we stress the importance of critical openness, plurality and cooperation for two pragmatic reasons. First, as the articles presented in this special issue suggest, the wider effects of medical and health-related knowledge, care, intervention, education and research are extensively and complexly distributed throughout social life, at a great variety of scales and through diverse spaces, temporalities, institutions, media, geographies and forms of government.^38^ The medical humanities is neither immune to nor separable from the influence of the life sciences. Minimally speaking, the practices that make up the medical humanities are deeply and irrevocably entangled in the vital, corporeal and physiological commitments of biomedical research.^39^ Sociologists of science such as Nikolas Rose and Maurizio Meloni have argued we live in times that are uniquely ‘biosocial’; human development, sociality, emotion and cognition, for example, are phenomena that cannot be understood via a fragmentary, parcellated account of discrete ‘biological’ and ‘sociocultural’ components.^40^ ^41^ The way that medical humanities has been defined in the past as operating as a more-or-less independent ‘actor’, mediating between service user/patient, clinician, educator or student, fails to recognise how medicine, illness and health are unevenly produced and distributed in contemporary society via complex assemblages of clinical and extra-clinical spaces.^21^

Second, the idea of the medical humanities having a ‘role’ to play within a wider research ecology presupposes the sanitary division of disciplines rather than the messy and mixed hybridities, collaborations and dilutions that underpin much of its work.^42^ As a heterogeneous and interdisciplinary set of practices—clinical, therapeutic, artistic, scholarly, activist and educational—the medical humanities stands to gain more from working through and indeed embracing the messy flexibility and inclusiveness gained from having no necessary or predetermined trajectory—particularly if it is to foster innovative research questions, be ready to revisit its norms and procedures of operation, as well as act as a counterweight to prevailing orthodoxies—than by assigning to itself a mantle by which to act.

## Critical collaboration and risk

A call for a critical medical humanities that prizes experimentation, reflexive practice, collaboration, and modes of sceptically risky thinking that are not easily wedded to a fixed role with regard to biomedicine, presents a number of challenges. Those challenges are faced by ourselves—researchers working under the restrictive intellectual and political economies of UK higher education—and by the humanities and social sciences as a whole, whose place in the ‘knowledge economy’ has been repeatedly, and for quite some time, characterised as one of crisis and emergency.^43–45^ Rather than taking up the language of crisis here, we want to focus on collaboration and assembly as key means through which the medical humanities can remain pervious to the domains that surround it—not least those domains described by the abstractions of biomedicine and social science. For if, to return to the second trajectory of the medical humanities that we outlined above, the humanities are called upon to ‘enable and promote fearless questioning of representations’, then might we not wonder how such fearless questioning would work to transform the medical humanities itself? How, in other words, might the medical humanities (as it draws upon history, philosophy, theology, literary studies, etc.) register—and indeed be deformed and transformed by—models of life, pathology and health that emerge from the biomedical and social sciences?

This special issue is characterised, then, by various kinds of collaboration and assembly. The papers that comprise it—whether sole- or co-authored—are marked by both strange and not-so-strange alliances and critical relationships out of which those papers’ arguments have emerged. Lynne Friedli and Robert Stearn's research on the operations of psychology within current workfare regimes, for example, draws on an intimacy with both the perpetrators of that logic and those subject to it, which is indebted to an interdisciplinary and tangled tactics of activism and scholarship (***see page 40–47***). Jan Slaby's programmatic articulation of the relationship between critical neuroscience and critical medical humanities draws on his immersion, as a philosopher, in interdisciplinary spaces, in which he has collaborated over many years with life scientists and social scientists (***see page 16–22***). And our own co-authored paper brings together three of us who not only tack back and forth between the humanities and the social sciences (sites of our ‘original’ disciplinary training) but spend much of our time in collaborative research with clinicians, life scientists and patients/service users.^46–48^ We recall Bruno Latour's well-known definition of the critic as ‘not the one who debunks, but the one who assembles. The critic is not the one who lifts the rugs from under the feet of the naive believers, but the one who offers the participants arenas in which to gather’.^33^

The special issue also brings together scholars who range widely in the foci of their concern as well as the scale at which their arguments find most vigorous purchase. All contributions take flight from sites other than that described as medical humanities’ ‘primal scene’—namely the encounter that enjoins a patient who has cancer and her clinician.^49^ We wanted explicitly to dislodge this mise-en-scène so as to foreground other kinds of relations, networks, nodes and entities through which health and medicine are made, and unmade. We wanted, additionally, to reflect on the scholarly and worldly resources that are not commonly captured within either medical humanities’ consolidating archive, or within its usual (inter)disciplinary machinery. Why, for example, have Continental philosophy (outside of a particular lineage of phenomenological thought),^50^ cultural theory, disability studies, queer theory, and science and technology studies been, hitherto, largely shadowy or eccentric presences, even as they have addressed, often extensively, problematics central to the medical humanities? What would a vigorous interrogation of the debates and subdisciplines that have tended to occupy centre stage within the medical humanities (we are thinking here of medical education, the history and philosophy of medicine, literature and medicine, and art therapies) reveal about that field's locus of concern, horizons of interest, and procedural norms?

## Experiments in critique

Each of the contributions to this special issue works with a different facet of the ‘critical’—whether via the deepening of its long philosophical histories, the torqueing of that history in new directions, or through explicitly bringing the energies of activism to bear on theoretical and policy-based problematics. In presenting this collection, we were eager, as intimated above, to extend an invitation to scholars addressing health, medicine and well-being who had not yet been drawn into debates within the medical humanities. This collection was not formed to produce a single message or dogma but to open what we hope will be a provoking and unusual set of insights, to inspire further research that is experimental and reflexive. With that in mind, we have invited short responses from a range of scholars, asking each to reflect on the central claims and potential limitations of each paper from his or her own often very different disciplinary vantage point, and as someone working more explicitly ‘within’ the medical humanities as it is currently constituted. We are delighted to include these critical commentaries and to be able to incorporate something of a call-and-response structure to experimental work that we feel deserves wider circulation and discussion. These commentaries not only provide an opportunity to overhear emerging debates between practitioners with very varied backgrounds but also give a sense of the stimulating, open and intellectually generous symposium that was held at Durham University at the end of 2013 and whose success has inspired this collection.

The first two papers presented here specifically address the role of critique in the medical humanities. Andrew Goffey examines the separation of different forms of knowledge production, the relative isolation of the humanities, social sciences and natural sciences, and the effects of this separation on the way that the language of immunology has permeated social, political and cultural arenas (***see page 8–13***). The theorisation of immunology in and beyond the life sciences has developed through the languages of warfare, and the penal and migration systems: following Foucault, much critical work has focused on how pathogens have been figured as ‘criminals’ or ‘foreign powers’, and the immune system likened to plainclothes police officers. This form of critique has attempted to wed biomedical research to a wider set of historical, economic and political conditions, in this case, the presentation of a biological mechanism is profoundly bound to 19th and 20th century forms of social, economic and political governance. But the act of critically highlighting the discursive nature of immunological thinking also has weaknesses that are pertinent to medical humanities projects whose critical insights depend on insisting that biomedical knowledge should be viewed as a social construction. Prime among these weaknesses is the superiority claimed by those whose critical perspective aims at exposing illusions, as well as the problematic equivalences drawn between a world of ‘constructions’ and the social practices said to govern them. Goffey, instead, warns against the hierarchies that criticism can produce and stresses the importance of avoiding the shortcuts upon which many of those hierarchies depend. This is an article that appeals to forms of medical humanities research that can be open about shared confusions and unresolved complexities.

Jan Slaby's paper on the role of critique and collaboration in approaches to neuroscience seeks a more sophisticated understanding of philosophy's role (***see page 16–22***). Slaby's work is important because he shows how concepts that are prevalent in the humanities are not necessarily ‘opposed to’ or constructed ‘outside of’ the material realities which are the traditional interests of the natural sciences. Rather than thinking of the work of the humanities and natural sciences as inhabiting intrinsically different epistemologies, Slaby is able to explain why negative critique must remain important within collaborative experimentation, but also why this cannot be the only viable avenue for collaboration. The medical humanities, it is argued, can learn much from perspectives formed in ‘critical neuroscience’ and its attempts to understand emerging kinds of technoscientific normativity—the commercial, legal, technological, ideational and epistemological trends that have come together to create and sustain the current widespread fascination with neuroscientific authority. Despite having made limited practical gains in terms of therapeutic applications, neuroscience has thrived thanks to its power to promise futures that are shored up by speculative venture capital and ‘big science’ solutions. Such a situation demands an analysis that can appreciate both what neuroscientists do and the wider contexts in which the brain, while invoked to naturalise complex behaviours, is recruited to actively shape new forms of subjectivity. Particularly important to this paper is how concepts of risk and probabilistic conceptions of illness have formed around the scientific promise heaped upon neuroscientific research, giving rise to the condition of being a ‘patient-in-waiting’ that is restricted by the ways health and agency can be defined by the marketplace. Slaby's synoptic approach is capable of observing the complex, long-term confluence of different factors that have shaped research in the natural sciences. What is crucial is how this paper manages the resources of a ‘critical neuroscience’ to draw from philosophy as well as diverse arenas of the social sciences.

In ‘Unpacking intoxication, racialising disability’, Mel Chen shows how concepts such as ‘toxicity’ and ‘intoxication’ are far from scientifically neutral (***see page 25–29***). Her development of a critical medical humanities approach to intoxication exemplifies the complex permeability of medicalised language that finds elaboration through discourses of economics, anthropology, human rights and colonial governance. In a manner that draws attention to the dispersed geographies and histories of where the ‘medical’ arises—with case studies ranging from contemporary economic crises in North America to the politics of opium trading and colonial authority in 19th century China—Chen argues that 21st century economics is saturated with the language of unhealthy or contaminated bodies. Since the banking crisis of 2008, ‘toxicity’ has been used to naturalise and give a body to economic disparities and to uphold a global developmental model that renders people, communities and environments ‘non-performing’ and thus invalid. Within this transnational context, toxic people, objects and communities are marked by a set of ‘incapacities’ whose non-performance is strongly predicated by norms of race and physical ability.

By illuminating the co-substantiating power of the ‘toxic’ with race and ability, Chen's aim is not to replace one sleek explanatory narrative with another but to explore and call attention to the alternative temporalities, chronologies and epistemologies of the intoxicated subject. This calls for a richer historical understanding of people deemed to be intoxicated—moving us beyond the individualising diagnostic criteria and therapeutic management of addiction and towards a thorough critique of standards of cognition, productivity, health and sociality. Resonant with work in critical disability studies,^51^ Chen's challenge to those working in the medical humanities is to appreciate how, through intoxication, we can interrogate the denigration of certain groups for their association with toxic states. She encourages us to be bold in occupying a paradoxical and potentially uncomfortable position: actually being intoxicated may in fact form an important critical site for resisting taken-for-granted dogmas that have sought to bind economic and medical well-being to a narrow set of functional norms.^52^ To expose what underpins and thus find alternatives to the accepted routines of the ‘rational’ adds critical awareness to medical humanities practitioners who seek a more plural set of alternatives, but may also be at odds with those who wish to streamline its intellectual and disciplinary codes. One of the important aspects of Chen's discussion of the medical humanities is to acknowledge the personal and professional risk of being critical.

Recent research in the medical humanities has stressed the embodied nature of experience, and this is often used as a way to counteract the numerical abstractions of the Western scientific imagination.^45^ This collection is cautious about these dichotomous engagements with scientific methods of inquiry and, more broadly, is keen to stretch conceptions of embodiment to accommodate the various ways that the human body can now be fragmented into discrete, commoditised units, and globally trafficked for profit. Tissues, organs, cells, DNA—all can be extracted, adapted, graded, disembodied and re-embodied. Bronwyn Parry's discussion of donors and surrogates in fertility treatments reflects on a particular aspect of this corporeal segregation and commercialisation (***see page 32–37***). Her article serves as an important reminder that a strong critique of neoliberal economics, which some argue coordinates new kinds of commercial exploitation, can risk ignoring the local cultural practices, material realities, motivations and experiences of clinical labourers. To show how clinical labour is not a homogenous category, Parry compares the living conditions of sperm donors in California and oocyte and sperm donors from Mumbai. There are important differences in terms of security, wealth and motivation between these two locations, and yet these distinctions are lost in those analyses that are focused solely on the systemic level or eager to stress the common victimhood of its protagonists. Moreover, where a critique of neoliberalism might expect to find impersonal forms of deregulation, contractualisation and internationalisation in the clinics of Mumbai, Parry finds a complex web of intermediaries, kin relations, gifting practices, and institutions eager to embrace regulation.

Parry's article carries important lessons for critical medical humanities scholarship: becoming critical of and developing a set of activist positions against a newly emerging clinical practice, especially if this practice is unevenly distributed on a global scale, does not necessitate a zero-sum game between argumentative force and explanatory detail. What Parry's ethnographically informed article highlights is the importance of local experience and styles of organisation, and how modern economies foster emerging medical practices in concert with older, longer-standing histories of social practice, uneven development and social inequality. The task of scholarship in this area is not to anticipate and reproduce a grand narrative of undifferentiated exploitation but to understand how clinical experience and capitalist enterprise, such as that of being a clinical donor or surrogate, develop within specific localities.

In the final contribution to this special issue, Lynne Friedli and Robert Stearn reflect on the activist edge of the critical medical humanities with an important theorisation and documentation of how psychological types and ideals, promoted by positive psychologists, have been mobilised by government employment schemes (***see page 40–47***). ‘Workfare’ is the name given to mandatory unpaid work carried out by social security claimants in the UK. Next to the long-standing reification of employment as a source of personal fulfilment and well-being, Friedli and Stearn provide disturbing evidence of how workfare programmes are supported by a form of psychological discipline, demanding that citizens take cognitive responsibility for their joblessness. Becoming a successful employee becomes a question of presenting the right psychological attitude or emotional orientation, rather than recognising that having a job is a complex consequence of economic and political policy or social disadvantage.

The medical humanities has made extensive interventions in the field of mental health, care and diagnosis, especially patient experience.^53^ ^54^ Friedli and Stearn's research, which draws conceptually and empirically from the wellsprings of their activism, shows how psychologists can be mobilised as political agents under the cover of professional neutrality. The recruitment of psychologists and the concomitant privatisation of public and economic policy upon the emotional lives of underprivileged sections of society are insidious processes. Not only does the critical medical humanities have a capacity to report on political practices that are, by their nature, obscured from public visibility, but Friedli and Stearn show how benevolent therapies are used to legitimise and make mainstream new forms of governance that aim to operate at the level of belief, emotion and affect.

## Concluding remarks

The papers and commentaries comprising this special issue of the journal set out a bold agenda for critical, collaborative and cross-disciplinary inquiry in the medical humanities. If Goffey, Slaby, Chen, Parry, Friedli and Stearn locate their work in fields adjacent to—if still outside—the traditional heartland of medical humanities scholarship, our task as editors has been to invite them to think through the implications of the methods, modes and sites of their analyses for scholars similarly concerned with questions concerning the complex making and re-making of medicine and health. And if, for readers, their work collectively challenges taken-for-granted ideas about the nature of critique, opens up productive avenues for further research, and raises uncomfortable and potentially irresolvable questions not just about the identity but about the capabilities and responsibilities of the medical humanities, then our hopes for this special issue will have been realised.

Work in the medical humanities has long pursued nuanced and reflective ways of analysing health, illness and medical care at varying sites and scales, in a wide range of historical and cultural contexts, through innovative forms of collaboration, and with diverse methods of enquiry. Embracing the complex role of critical collaborator—a role based on notions of entanglement, rather than servility or antagonism, and so reflexively constituted and reworked—will, we suggest, enrich and develop the imaginative and creative heterodox qualities and practices which are the field's core strengths.
